# Health service capacity of rheumatology and immunology departments in China: A cross-sectional study of young and middle-aged physicians

**DOI:** 10.1515/rir-2026-0018

**Published:** 2026-07-13

**Authors:** Lin Qiao, Yan Geng, Ling Zhao, Chanyuan Wu, Yingqian Mo, Zhu Chen, Xiangfeng Xu, Lihua Duan, Yi Zhao, Jiuliang Zhao, Qian Wang, Yan Zhao

**Affiliations:** Department of Rheumatology and Clinical Immunology, Peking Union Medical College Hospital (PUMCH); Chinese Academy of Medical Sciences & Peking Union Medical College; National Clinical Research Center for Dermatologic and Immunologic Diseases (NCRC-DID), Ministry of Science & Technology; State Key Laboratory of Complex Severe and Rare Diseases; Key Laboratory of Rheumatology and Clinical Immunology, Ministry of Education, Beijing, China; Department of Rheumatology and Clinical Immunology, Peking University First Hospital, Beijing, China; Department of Rheumatology, The First Hospital of Jilin University, Changchun, Jilin Province, China; Department of Rheumatology, Sun Yat-Sen Memorial Hospital, Sun Yat-Sen University, Guangzhou, Guangdong Province, China; Department of Rheumatology and Immunology, The First Affiliated Hospital of the University of Science and Technology of China, Division of Life Sciences and Medicine, University of Science and Technology of China, Hefei, Anhui Province, China; Department of Rheumatology, Zhoushan hospital, Zhoushan, Zhejiang Province, China; Department of Rheumatology and Clinical Immunology, Jiangxi Provincial People’s Hospital, The First Affiliated Hospital of Nanchang Medical College, Nanchang, Jiangxi Province, China; Department of Rheumatology and Immunology, West China Hospital, Sichuan University,; Clinical Institute of Inflammation and Immunology, Frontiers Science Center for Disease-related Molecular Network, West China Hospital, Sichuan University, Chengdu, Sichuan Province, China

**Keywords:** health service capacity, rheumatology and immunology departments, young and middle aged physicians

## Abstract

**Background and Objectives:**

Rheumatic diseases are among the most prevalent chronic health conditions worldwide. This study aimed to systematically assess the diagnostic, therapeutic, and service capabilities of rheumatology and immunology departments across China.

**Methods:**

A questionnaire-based survey was conducted among young and middle-aged rheumatologists from May to November 2023. The questionnaire collected data on demographics, departmental infrastructure, and service capabilities.

**Results:**

A total of 2047 rheumatologists from 934 hospitals completed the survey. Among the participating hospitals, 817 (87.5%) were tertiary institutions. Most respondents (1970/2047, 96.5%) reported that their hospitals had rheumatology and immunology departments. With respect to the departmental scale, 65.0% of the respondents reported having 20–50 inpatient beds, and 39.0% (603/1546) of the departments had 6–10 physicians. Only 33.8% (691/2045) of the hospitals were capable of performing basic immunologic blood tests. The availability of key modalities was as follows: dual-energy CT (60.6%, 1238/2044), musculoskeletal ultrasound (87.0%, 1778/2044), sacroiliac joint CT (90.9%, 1857/2044), labial gland biopsy (81.3%, 1662/2044), renal biopsy (80.6%, 1647/2044), and synovial biopsy (62.6%, 1280/2044). Most surveyed physicians used Western medicine for patient treatment. In addition, 89.0% (1806/2030) of the respondents reported conducting follow-up for patients with chronic rheumatic disease.

**Conclusions:**

Rheumatology is among the fastest-growing specialties. Our survey highlights the need to enhance public awareness, optimize specialist training, and strengthen healthcare delivery and academic research. With increasing national attention, we anticipate considerable advancements in the fields of rheumatology and immunology in China.

## Introduction

Rheumatic diseases are among the most common chronic health problems worldwide, imposing a substantial burden on patients’ quality of life and generating considerable societal costs related to healthcare utilization, work loss, disability and mortality. China, with a population of approximately 1.4 billion, is a developing country in East Asia where rheumatology and immunology is a relatively young field. The discipline began with the establishment of the first rheumatology and immunology department at Peking Union Medical College Hospital in 1980. Over the past few decades, substantial progress has been made. The Chinese Rheumatology Association (CRA) was founded in 1985, followed by the Chinese Rheumatology Physician Association in 1999. These professional organizations strongly support disciplinary development. Since 2009, the Chinese SLE Treatment and Research Group (CSTAR) and the Chinese Rheumatism Data Centre (CRDC) have provided valuable research data through large-scale platforms, facilitating multicentre investigations.

It is estimated that more than 100 million patients in China have rheumatic and immune diseases.^[1,2]^ According to the fourth national survey, as of September 30, 2018, there were 12,189 rheumatology professionals in China—a 1.7-fold increase compared with 2015. The number of rheumatology and immunology departments reached 3372 (a 1.9-fold increase), and the number of independent departments increased 1.6-fold from 737 to 1180. Despite this growth, a major challenge remains the considerable imbalance between patient demand and available rheumatologists. Furthermore, many patients are not in clinical remission or require specialist-led care, resulting in considerable unmet needs.

This study is primarily based on a survey of young and middle-aged physicians and aims to assess the diagnostic, therapeutic, and service capabilities of rheumatology and immunology departments across China. The findings are intended to provide empirical insights for the discipline’s high-quality development and to identify current barriers and challenges.

## Method

To promote the development of the rheumatology and immunology discipline, the Youth Committee of the CRA has taken the lead in conducting a nationwide survey. The study was approved by the Ethics Committee of Peking Union Medical College Hospital. Eligible participants were young and middle-aged physicians under 60 years of age, with a small number of physicians aged approximately 60 years also included. Data were collected using a structured questionnaire (supplement files), which covered respondents’ general information; the establishment of rheumatology and immunology departments in their hospitals; staffing and bed allocation; the current status of diagnostic approaches for rheumatic diseases; and the practice of chronic disease management, follow-up and patient referral.

From May to November 2023, a total of 2500 questionnaires were distributed to rheumatologists *via* the online platform of the China Association for the Promotion of Human Health Science and Technology, and 2047 valid questionnaires were collected. Completion and return of the questionnaire implied informed consent.

The data were analysed using SPSS 27.0. The qualitative data are presented as frequencies and percentages, whereas the quantitative data are presented as the means.

## Results

### Basic Information

**Figure 1:**
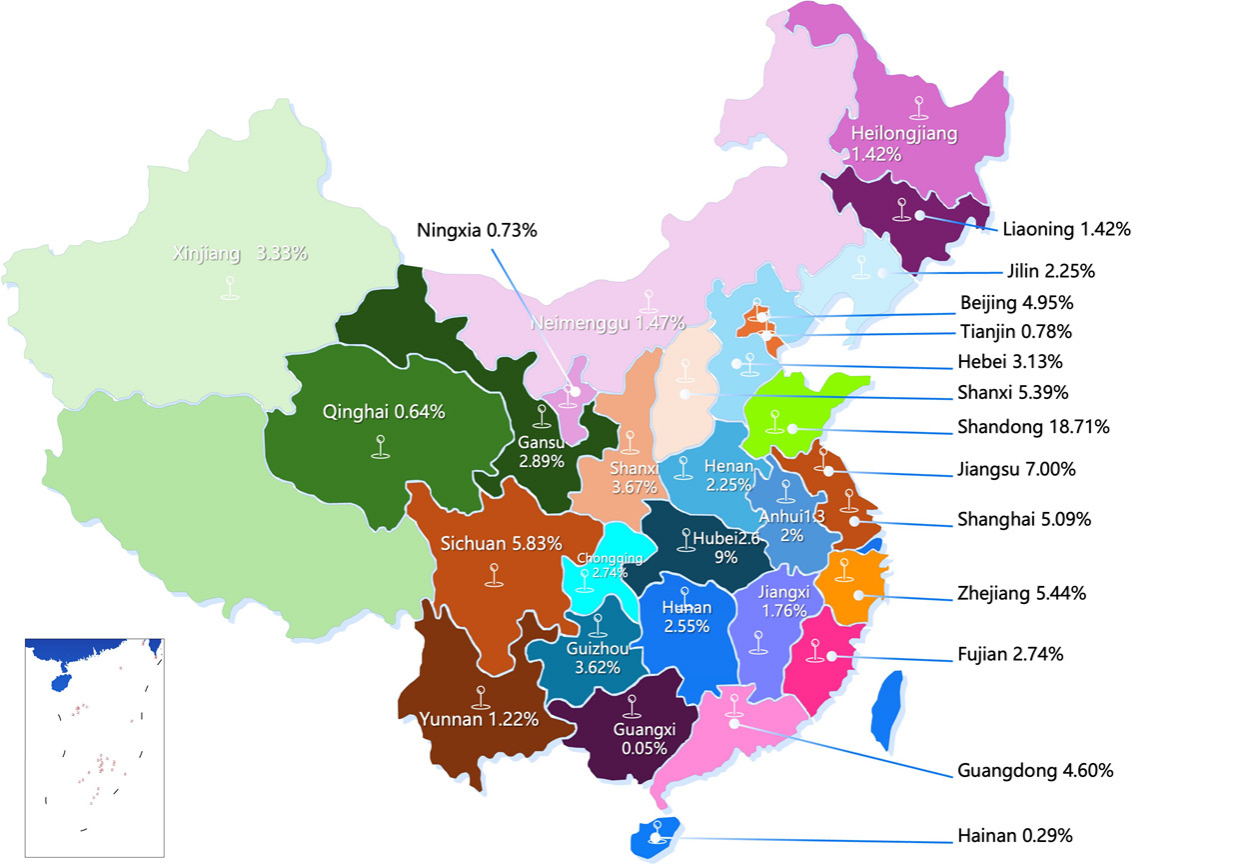
Distribution of the respondents across the country (N = 2047).

A total of 2047 rheumatologists from 934 hospitals in 30 provinces participated in the survey. The geographical distribution of the respondents is shown in Figure 1. Most respondents were from eastern China (859/2047, 42.0%). Shandong (18.7%, 383/2047), Jiangsu (7.0%, 143/2047), and Sichuan (5.8%, 119/2047) were the top three provinces. Chinese hospitals are classified into tertiary, secondary and primary hospitals on the basis of their service scope, size, and technical capability. A total of 87.5% (817/934) of the respondents were from tertiary hospitals, 12.2% (114/934) were from secondary hospitals, and 0.3% (3/934) were from primary hospitals.

Among the respondents, 66.2% (1353/2044) were female, and 33.8% (691/2044) were male (F: M ratio of 1.96: 1). A total of 74.3% (1519/2044) of the participants were aged 30–49 years, and 20% (409/2044) were 50–59 years old. With respect to education, 31.33% (641/2046) held bachelor’s degrees, 49.71% (1017/2046) held master’s degrees, and 18.57% (380/2046) held doctoral degrees. Senior physicians (deputy chiefs and chief physicians) accounted for 64.32% (1316/2046), while junior professionals accounted for 4.6% (94/2046).

### Departmental Configuration

Most respondents (1970/2047, 96.5%) reported having rheumatology and immunology departments (including those merged with haematology, nephrology, or endocrinology), and 94.7% (1865/1970) were from tertiary hospitals. In hospitals without standalone rheumatology departments, patients were typically managed by traditional Chinese medicine (*N* = 30), general internal medicine (*N* = 15), or orthopaedics (*N* = 9) specialists.

**Figure 2:**
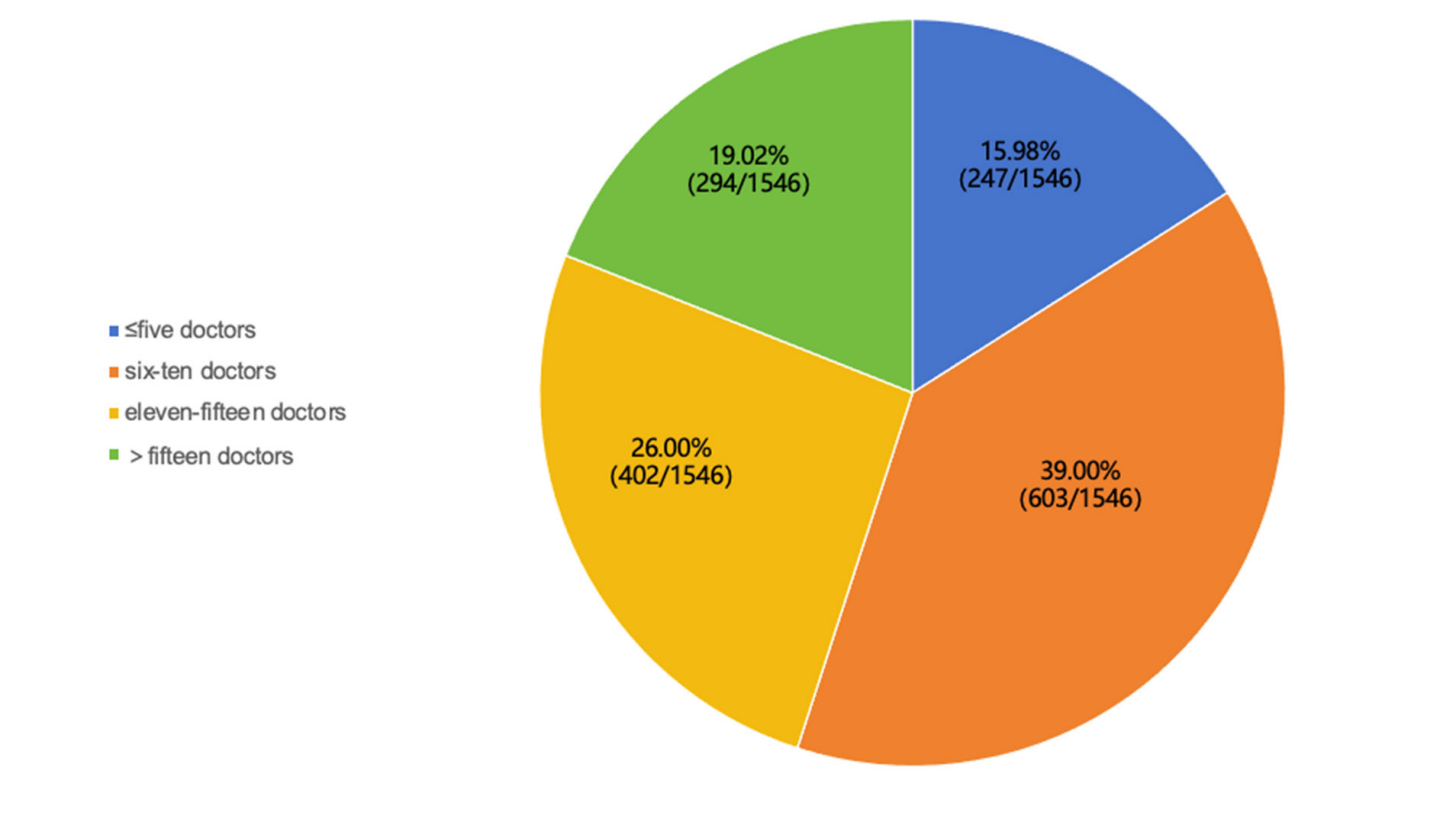
Number of respondents in independent rheumatology and immunology departments (N = 1546).

Among hospitals with rheumatology and immunology departments (represented by 1, 546 respondents who completed the questionnaire), 65.0% had 20–50 beds, 17.0% had > 50 beds, and 18.0% had < 20 beds. With respect to physician numbers, 39.0% (603/1546) of departments had 6–10 physicians, 19.0% (294/1546) had ≥ 15, and 16.0% (247/1546) had ≤ 5 (Figure 2).

### Diagnostic and Evaluation Techniques

**Figure 3:**
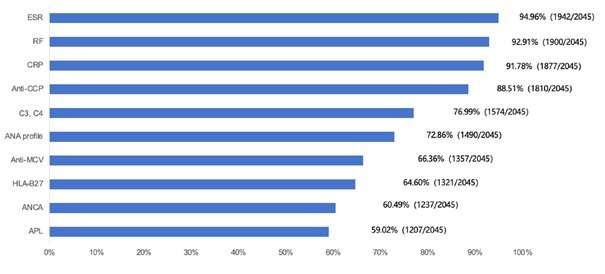
Blood test items (N = 2045). ESR, erythrocyte sedimentation rate; RF, rheumatoid factor; CRP, C-reactive protein; Anti-CCP, Anti-cyclic citrullinated peptide antibody; ANA, antinuclear antibody; Anti-MCV, anti-mutated citrullinated vimentin antibody; HLA, human leukocyte antigen; ANCA, anti-neutrophil cytoplasmic antibody; APL, antiphospholipid antibody.

Only 33.8% (691/2045) of the respondents’ hospitals could perform all the basic immunologic blood tests, including tests for inflammatory markers and specific autoantibodies (Figure 3).

**Figure 4:**
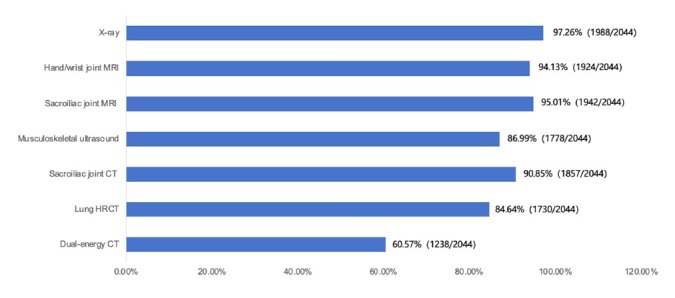
Imaging examination (N = 2044). MRI, magnetic resonance image; CT, computed tomography; HRCT, high resolution computed tomography.

With respect to imaging examinations (Figure 4), the availability was as follows: dual-energy CT (60.6%, 1238/2044), musculoskeletal ultrasound (87.0%, 1778/2044), and sacroiliac joint CT (90.9%, 1857/2044). Additionally, 78.5% (1605/2045) of the hospitals could analyse synovial fluid, 42.8% (875/2045) could perform polarized light microscopy, and 62.6% (1280/2044) were capable of synovial biopsy. The proportions of hospitals performing labial gland biopsy and renal biopsy were 81.3% (1662/2044) and 80.6% (1647/2044), respectively.

### Treatment Options

In terms of oral medications, most surveyed respondents used Western medicine to treat rheumatic diseases. Although traditional Chinese medicine (TCM) has a deep historical foundation in China, only 2.3% (47/2043) of the respondents used TCM alone, while 40.9% (836/2043) used an integrative approach combining Chinese and Western medicine. A high proportion of hospitals offered orthopaedic surgical treatment (72.2%, 1443/1998) and performed surgeries for rheumatoid arthritis (*e.g*., synovectomy, arthroscopy), and 64.7% (1292/1998) performed surgeries for spondyloarthrits and osteroarthrits. Furthermore, 1109 respondents reported the ability to provide joint arthroplasty services.

### Follow-up Approaches

**Figure 5:**
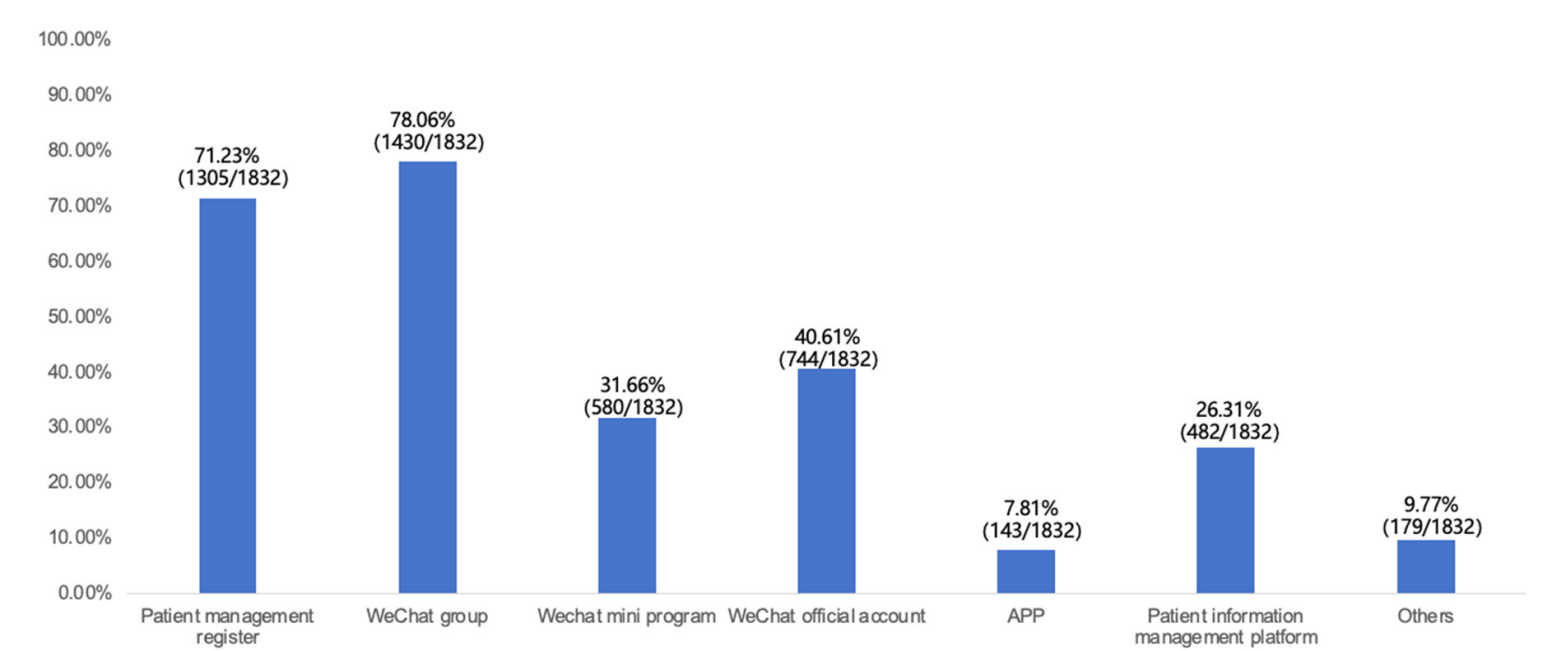
Patient management platform (N = 1832).

Most respondents (89.0%, 1806/2030) reported managing and following up patients with chronic rheumatic disease. However, only 40.8% (833/2042) had access to information-based chronic disease management platforms within their hospital. Beyond hospital platforms, 78.1% (1430/1832) used WeChat groups for follow-up, 71.2% (1305/1832) used registration books, and only 7.81% (143/1832) used mobile applications (Figure 5). Among 850 respondents, 77.4% (658/850) reported that chronic disease management was integrated with the hospital system. The most commonly used online service features were appointment registration (88.5%, 807/912), patient health education (85.7%, 782/912), and medical records establishment (77.0%, 702/912). Additionally, 37.9% (676/1784) used information platforms for patient referrals. However, 25.9% (220/850) reported difficulties in contacting patients *via* these platforms. Many respondents indicated that existing platforms lacked clinically necessary functions.

## Discussion

China has a large population and strained healthcare resources. According to the 2023 China Health Statistics Yearbook, tertiary hospitals accounted for 9.5% (3523/36,976) of all hospitals, secondary hospitals accounted for 30.1% (11,145/36,976), and primary hospitals accounted for 34.7% (12,815/36,976). Compared with 2019, the number of tertiary hospitals increased by 28.2%, whereas the numbers of secondary and primary hospitals increased by 15.1% and 13.8%, respectively. These findings indicate a more rapid expansion of tertiary hospitals.

In the present survey, the participants were from 934 hospitals nationwide, including 817 (87.5%) tertiary hospitals, 114 (12.2%) secondary hospitals, and 3 (0.3%) primary hospitals. This skewed distribution may indicate that tertiary hospitals ofer better professional platforms, training opportunities, and career prospects for young rheumatologists. In contrast, many secondary and primary hospitals lack well-established rheumatology departments and supportive practice environments, leading to a concentration of young physicians in tertiary centres. Consequently, our findings may largely reflect the current status of rheumatology care in tertiary hospitals.

Rheumatology developed much later in China than in Western countries, where the rheumatologist-to-population ratio ranges from 1: 40,000 to 1: 60,000.^[3,4]^ Like patients in other developing countries,^[5]^ patients in China often seek rheumatic disease care directly at tertiary centres, partly because primary care training in rheumatology is inadequate. Since 2019, China’s National Health Commission has recommended the establishment of independent rheumatology and immunology departments in tertiary hospitals. In 2019, only 41.1% (1129) of the tertiary hospitals had such specialized departments. The current number of rheumatologists is unclear. In a hierarchical healthcare system, patients are overly concentrated in high-level comprehensive hospitals. A 2009 nationwide workforce survey reported an average of only 1.67 rheumatologists per million people,^[6]^ highlighting the heavy workload of specialists. Given the growing gap between supply and demand in the rheumatology workforce, strengthening rheumatology services at hospitals of all levels is crucial for better resource utilization.

China’s population distribution is uneven, with higher density and greater medical resources in eastern and coastal regions. Independent rheumatology and immunology departments were established earlier in major municipalities and provinces, such as Shandong, Guangdong, Zhejiang, and Jiangsu, attracting more specialists. In contrast, some western and southern provinces lag behind. The geographical distribution observed in this survey, with the greatest number of rheumatologists in eastern China, confirms this developmental imbalance, which is also seen in the U. S. and Australia.^[7]^ Remoteness is associated with increased mortality in patients with chronic diseases, emphasizing the need to overcome geographic barriers to care. E-rheumatology and telemedicine offer promising solutions by facilitating remote consultations and improving access for patients in under-served areas.

Most practising rheumatologists in China are female (F/M ratio of 1.96:1). While multiple factors influence career choice,^[8,9]^ the ambulatory nature of rheumatology may lead to better work-life balance. This specialty involves the management of numerous complex diseases, and China now has a growing number of well-educated rheumatology professionals. Our study focused on young and middle-aged specialists, with 74.3% aged 30–49 years. Most respondents were from tertiary hospitals, 68.28% held postgraduate degrees, and 65.31% were senior professionals, indicating that young and middle-aged physicians form the backbone of rheumatology and immunology departments in tertiary hospitals. Over the past decade, government research funding has increased substantially,^[10]^ providing greater support for young rheumatologists and encouraging specialization in both clinical practice and research.

Rheumatic diseases are diverse and heterogeneous. The CSTAR and CRDC databases include more than 180,000 patients, covering more than 30,000 patients with lupus erythematosus; 70,000 patients with rheumatoid arthritis; and more than 20 other conditions, such as scleroderma, takayasu arteritis, psoriatic arthritis, and gout.^[11,12]^ Diagnosis relies on patient history, physical examination, laboratory tests, imaging, and pathology. Reliable objective examinations are therefore essential. Our results show that specific immune markers (*e.g*., APL, ANCAs, HLA-B27, and anti-CCP) are available in fewer than 70% of hospitals and that the rates of pathology and imaging examinations vary widely, indicating uneven diagnostic capabilities across China.

Over the past two decades, Chinese rheumatology has advanced rapidly in clinical practice. Nationwide registries such as CSTAR and CRDC have characterized Chinese patient populations and informed clinical policies. To standardize clinical practice, the CRA has developed guidelines covering 23 rheumatic diseases, including SLE, RA, AS, pSS, and rarer conditions such as PMR, with the goal of reducing misdiagnoses and improving practice quality. Moreover, pharmaceutical regulations in China have become more liberalized. Data on newer drugs^[13,14]^ and domestically developed biologics^[15,16]^ are becoming increasingly available. Although TCM has a long history in treating rheumatic diseases—for example, Tripterygium wilfordii Hook F (TwHF) has been used to treat RA since the 1960s^[17]^ —our data indicate that Western medicine remains more commonly used in clinical practice.

Rheumatology is well positioned to influence the future of chronic disease care. Our research revealed that in most tertiary hospitals, physicians engage in chronic disease management and follow-up, but only 40.79% use a dedicated digital management platform. Many rely on WeChat groups, phone calls, or paper records. Referral systems in primary, secondary and tertiary care are underdeveloped. Challenges include a shortage of rheumatologists and limited support from specialist nurses, occupational therapists, and physiotherapists. Telemedicine could help address gaps outside urban centres, with evidence supporting its benefits for rural patients despite existing implementation barriers.^[18]^

Beyond medical treatment, China emphasizes patient-centred care and support. Patient support groups and associations provide psychological counselling, disease education programs, and social support to help patients cope with long-term challenges. Social media platforms can aid research, education, and professional networking in rheumatology. Mobile health (mHealth) technologies enhance patient engagement, monitoring, and outreach, improving medication adherence and follow-up. Empowering patients in health education, rehabilitation, and self-monitoring is beneficial. Furthermore, artificial intelligence (AI) holds great potential for patient management and follow-up by improving treatment efficiency, personalizing treatment, and reducing costs, although issues such as privacy protection and technological dependence must be addressed. With ongoing technological advancements, the application of AI in medicine will likely expand.^[19]^

## Conclusion

Although rheumatology is a relatively new specialty in China, it is among the fastest growing. Considerable challenges and disparities remain, but opportunities for progress are substantial. Continued efforts are needed to raise public awareness, improve training, and enhance healthcare delivery and research. We believe that rheumatology will become one of the most promising clinical fields in China, with rapid advancements expected in clinical practice, research and international recognition.
